# diArk 2.0 provides detailed analyses of the ever increasing eukaryotic genome sequencing data

**DOI:** 10.1186/1756-0500-4-338

**Published:** 2011-09-09

**Authors:** Björn Hammesfahr, Florian Odronitz, Marcel Hellkamp, Martin Kollmar

**Affiliations:** 1 Abteilung NMR basierte Strukturbiologie, Max-Planck-Institut für Biophysikalische Chemie, Am Fassberg 11, D-37077 Göttingen, Germany

## Abstract

**Background:**

Nowadays, the sequencing of even the largest mammalian genomes has become a question of days with current next-generation sequencing methods. It comes as no surprise that dozens of genome assemblies are released per months now. Since the number of next-generation sequencing machines increases worldwide and new major sequencing plans are announced, a further increase in the speed of releasing genome assemblies is expected. Thus it becomes increasingly important to get an overview as well as detailed information about available sequenced genomes. The different sequencing and assembly methods have specific characteristics that need to be known to evaluate the various genome assemblies before performing subsequent analyses.

**Results:**

diArk has been developed to provide fast and easy access to all sequenced eukaryotic genomes worldwide. Currently, diArk 2.0 contains information about more than 880 species and more than 2350 genome assembly files. Many meta-data like sequencing and read-assembly methods, sequencing coverage, GC-content, extended lists of alternatively used scientific names and common species names, and various kinds of statistics are provided. To intuitively approach the data the web interface makes extensive usage of modern web techniques. A number of search modules and result views facilitate finding and judging the data of interest. Subscribing to the RSS feed is the easiest way to stay up-to-date with the latest genome data.

**Conclusions:**

diArk 2.0 is the most up-to-date database of sequenced eukaryotic genomes compared to databases like GOLD, NCBI Genome, NHGRI, and ISC. It is different in that only those projects are stored for which genome assembly data or considerable amounts of cDNA data are available. Projects in planning stage or in the process of being sequenced are not included. The user can easily search through the provided data and directly access the genome assembly files of the sequenced genome of interest. diArk 2.0 is available at http://www.diark.org.

## Background

The International Human Genome Project needed almost 13 years for the sequencing of the first human genome [1]. While Celera, using the same Sanger technique, already accelerated human genome sequencing to three years by applying a whole genome shotgun instead of the primer based approach [2], the sequencing of even the largest mammalian genomes has become only a matter of days with current next-generation sequencing methods [3]. The bottleneck for providing the analysis of a eukaryotic genome is thus not the sequencing process anymore [4]. The most time consuming part is the assembly and even more the annotation of genes, RNA, and other genetic features [5]. Nevertheless, while only a few genome assemblies have been made public per year at the beginning of the century, dozens of genome assemblies are released per month today. A further increase in the speed of releasing genome assemblies may be expected because of the increasing number of next-generation sequencing machines worldwide [6], together with the announcement of major sequencing plans (see for example the 1000 human genomes project [7], the 1001 arabidopsis genomes project [8], the 1,000 Plant & Animal reference genomes project [9], and the 10,000 vertebrates genomes project [10]).

There are many steps to produce a complete and gap-less genome sequence of an organism. First draft versions often contain sets of so-called contigs that have been built from the assembly of whole genome shotgun reads. The genome coverage is the most important factor determining contig length. In the following steps during the assembly process the contigs are organised into supercontigs and finally into chromosomes. In the finishing process, gaps are filled by direct sequencing of the corresponding regions. However, the publication of the genome sequence of an organism does not correlate with the status of the assembly process. Some genome assemblies have been published although they are very fragmented and represent rather early draft assemblies (e.g. [11-14]), while finishing and gap-closing have already been done for other genomes still waiting to be published. It is obvious that analyses based on genes, genomic regions, or proteins need high coverage genome sequences and assemblies to very long contigs or even supercontigs. This is especially true for the analysis of genes of higher eukaryotes that are often spread over hundred thousands of base pairs.

How can a researcher find out which organisms have already been sequenced, how good the quality of the latest assembly is, and what the differences between the sometimes many different assemblies of the same genome are? To provide access to genome data, five major databases have been developed: GOLD [15], NCBI Genome Project (will soon be reorganized into NCBI BioProject) [16], National Human Genome Research Institute (NHGRI) [17], International Sequencing Consortium [18], and diArk [19]. The GOLD database monitors finished and ongoing genome and metagenome sequencing projects of all branches of the tree of life [15]. The largest part of the database is related to prokaryotes for which most of the about 130 metadata fields have been designed. GOLD's strength therefore is the listing of the prokaryotes, while it is outdated for eukaryotes. For example, GOLD announces 156 eukaryotes as published (although several of these are listed as "unpublished" in the table, status: March 10, 2011) while genome assemblies of 358 eukaryotes have been published according to diArk (status: March 10, 2011). The NCBI Genome Project pages list all sequencing centres participating in a certain sequencing project and provides many links to other species resources (species databases, BLAST and genome browser pages, publications, etc.). However, the list of these projects is far from being up-to-date. Here, 431 eukaryotes are available and listed as complete or draft assembly, while diArk provides assemblies for 613 species. The NHGRI hosts a list of approved sequencing targets (almost exclusively eukaryotic) with limited additional information. However, most eukaryotic projects are not listed, and the project status (not started, in process, complete) is often not up-to-date. For example, the sequencing of *Geomyces destructans *is still listed as "not started" although a very good draft assembly is already available. The International Sequencing Consortium hosts a list of comparable information to the NHGRI.

diArk 2.0 is the most up-to-date database for eukaryotic sequencing projects, providing in the latest version many meta-data like sequencing and read-assembly methods, sequencing coverage, GC-content, extended lists of alternatively used scientific names and common species names, and various kinds of statistics. diArk only lists those projects, for which genome assemblies or considerable amounts of cDNA data are available. diArk does not list projects that are planned, and does not track the various stages of the genome sequencing process (species targeted, awaiting DNA, DNA library prepared, etc.) as it is done by GOLD [15]. Due to the next-generation sequencing methods sequencing has become so fast and cheap that the time frame between planning and finishing sequencing projects is in the order of weeks and not years anymore. Although independent groups have not sequenced too many identical species yet, sequencing has started to become competitive so that project plans are often not announced anymore and finished sequences claimed by press releases [20]. The virtue of the sequencing projects is the data, and thus the intention of diArk is to provide easy and fast access to where and which eukaryotic data may be obtained.

## Methods

### The technologies

The system is running on Linux. The database management system is PostgreSQL [21] supported by pgpool-II [22]. The web application framework is Ruby on Rails [23], which is based on the object orientated programming language Ruby [24]. In order to present the user with a feature rich interface while minimizing the amount of transferred data the site makes extensive use of modern Web 2.0 techniques like Ajax (Asynchronous JavaScript and XML) using Prototype [25], and Lightwindow [26]. Graphs are drawn using the graphical toolkit Protovis [27,28], the statistical programming language R [29], and SVG [30]. Ruby together with BioRuby [31] is also used for scripts that automatically retrieve data via the NCBI-API, reconstruct the phylogenetic tree of diArk's species, and analyse genome assembly files. All technologies used are freely available and open source.

### The database

**Figure 1:**
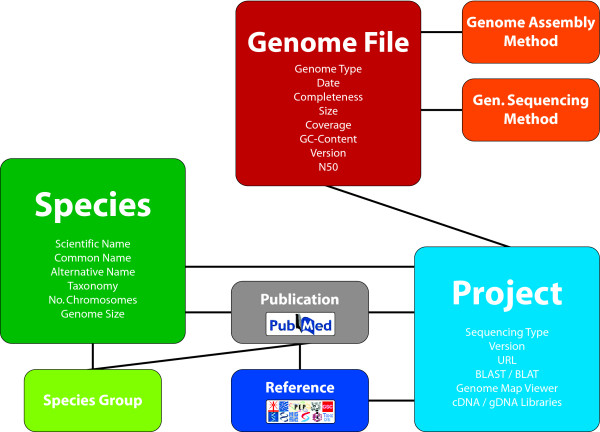
**Schematic organisation of the database**. The diagram shows the major tables of the database and their connections. Some of the content fields of the three main tables Species, Project, and Genome File are listed. Details to publications are obtained from NCBI via their API. The References table contains the major sequencing centres and species project web pages.

diArk has been developed with a custom database schema due to the unique requirements of the system [19]. Initially, three interconnected tables had been at the centre of the database: species, projects, and publications. This basic concept has significantly been extended by more than doubling the number of database tables and by increasing the number of fields in existing tables (Additional file 1). Most importantly, a table for genome file data has been added to which several further tables are connected representing sequencing and assembly methods (Figure 1, Additional file 1).

The genome file table contains information about genome assemblies. Genome assembly files are retrieved from sequencing centres, dedicated species/taxa sequencing pages, or from the NCBI database. While some information is directly calculated from the assembly files, other information is manually added to the genome file table. Every assembly file gets a genome type identifier based on the fasta-entries. The most important genome types are Chromosome, Uchromosome (these files contain contigs/supercontigs, which could not be mapped to any (unknown chromosome) or anchored (random chromosome) to a certain chromosome), Supercontigs, Contigs, Ureads (unplaced reads), Apicoplast, Chloroplast, Kinetoplast, and Mito (mitochondrial DNA). In addition, there are some special extensions to the file types, for example "assembly1", "assembly2", etc.. These extensions indicate that different assemblies for the same genome are available. For example, if assemblies were produced from different sequencing data like in the case of *Drosophila pseudoobscura *(assembly1: [32]; assembly2: unpublished assembly of The Institute for Genomic Research) or if the same reads were assembled using different methods/software like in the two *Bos taurus *genome assemblies (assembly1: [33]; assembly2: [34]).

If possible, the version of the assembly as well as the release date of the data is provided. In general, the versions and release dates are entered manually as given by the sequencing centres. Otherwise the dates are used at which the files were saved in the ftp-directories. For NCBI-assembly data, we store the dates at which the data has been submitted to NCBI. Please note that the version numbers do not correlate among sequencing centres and NCBI. Also, we rank the completeness of the genome assemblies as a rough estimate of the quality of the data. If provided by the sequencing centres, the genome coverage of the assembled sequence data is given. For some assemblies, comments are written that provide further background information about differences to earlier assemblies and problems during the assembly process, for example.

**Figure 2:**
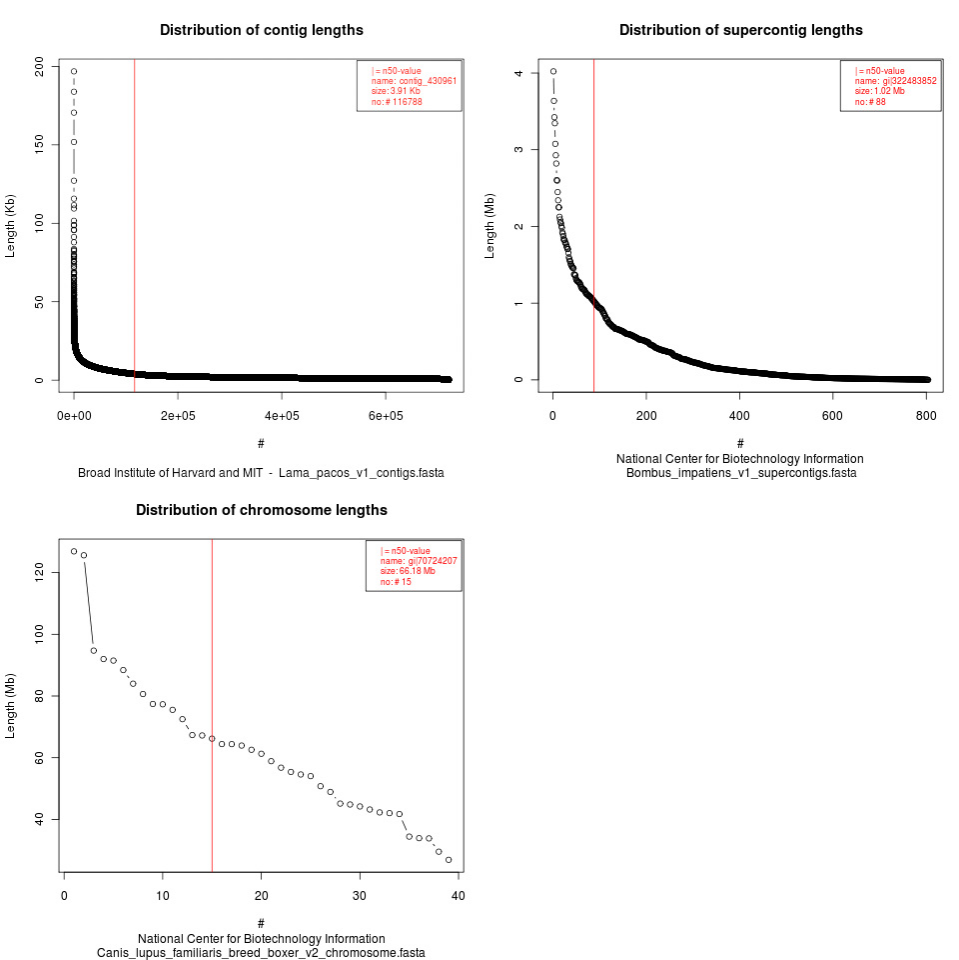
**Contig distribution for three sample genome assemblies**. A) Example of a low-coverage mammalian genome. B) Example of a high-coverage insect genome. C) Example of a chromosome assembly. All chromosomes are plotted as separate entries.

In addition to this manually collected information, the GC content, the size in Giga-base-pairs, the number of fasta-entries, the occurrence of illegal characters in the sequences (not being g/G, a/A, t/T, c/C, or n/N), and the N50 of the assemblies are calculated from the fasta files. The N50 value is a measure of contig length and is calculated by adding up contig lengths starting with the longest contig. The length of that contig, which leads to at least half of the assembly, is the N50 value. The longer the contigs are the longer is the contig that overcomes the half-genome barrier. All contig lengths are counted and plotted in decreasing length together with the N50 value (Figure 2). These graphs provide additional information to the user to judge the quality of the assembly. Accession numbers are only stored from NCBI data.

For every genome file the sequencing methods and the assembly software were collected, if available. The next-generation sequencing methods strongly differ in their usefulness concerning de-novo assemblies, and therefore this information together with the sequencing coverage and the library types used for sequencing is absolutely essential to judge the quality of the data.

### The web interface

**Figure 3:**
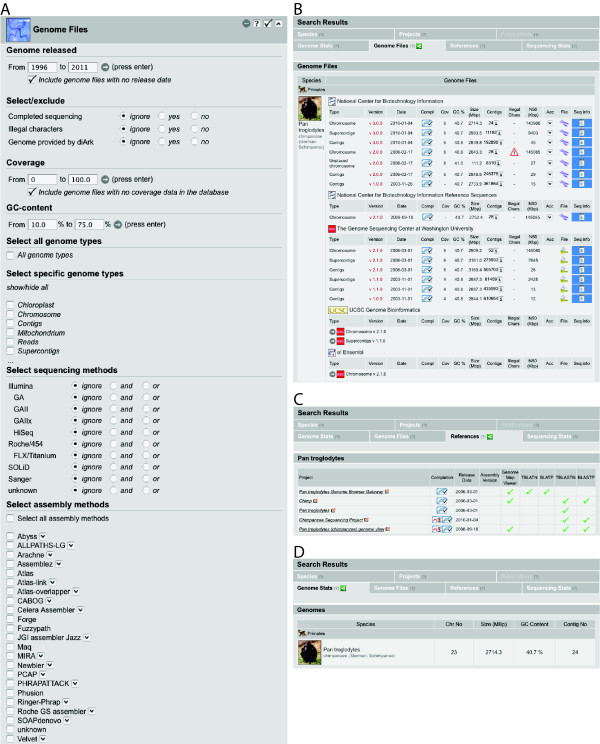
**Screenshots of diArks "Genome Files" search module and several result views**. A) The new "Genome Files" search module of diArk allows a detailed search for species that were sequenced with a specific sequencing method, for certain assembly methods, for specific genome types, for the completeness of the assembly, for illegal characters (not a/A, t/T, g/G, c/C, n/N), and for genomes provided by diArk. Furthermore, the data can be filtered by the GC-content, by the sequence coverage, and the release date of the genome assemblies. B) The "Genome Files" result view provides an overview about the different genome assemblies generated by the sequencing centres. Clicking on the symbols provides further details and the possibility to download the genome file. C) The "References" result view provides an overview about some data analysis options the species project pages offer, like BLAST pages or access to genome browsers. D) The "Genome Stats" result view gives a species based overview about several genome statistics, like the chromosome numbers and the GC-contents, with the species ordered according to their taxonomy so that closely related organisms can be compared.

The web interface always represents the current state of the database, and all tables and graphs are calculated on-the-fly depending on users requests. The database is searched using any of the six *search modules*, or a combination of them. We have added a new module, called "Genome Files", for searching the data content of the genome file table and associated tables (Figure 3A). The results of the search can be browsed in *result views*. Previously, three result views had been offered, the "Species", the "Publications" and the "Projects" result view. The new "Genome Stats" result view provides a fast overview of important genome characteristics in direct comparison of evolutionarily related species and includes chromosome numbers (if known), genome sizes (as calculated from the assembly files, given as number of base pairs included in the chromosome-, supercontigs-, or contigs-file, in descending priority), the GC-contents, and the number of contigs (Figure 3D). The "Genome Files" result view provides a direct comparison of the data related to the assembly files (Figure 3B). Here, data as provided from NCBI and the sequencing centres can be downloaded (in accordance with the Bermuda principles and the Ford Lauderdale agreement [35]) and the graphs presenting the size distribution of the contigs/supercontigs/etc. can be viewed (Figure 2). The "References" result view provides information about tools and material as provided by the species sequencing pages, for example, whether certain species homepages provide BLAST search possibilities or access to genome browsers (Figure 3C). The "Sequencing Stats" result view provides many graphs presenting various aspects of the data (in total or according to the selection by the user; see also below).

In addition to the modular search, which allows a powerful and very detailed definition of the search, diArk provides a "Fast Search" just offering the main search options: the search for a single species, the selection of model organisms or given taxa, the selection for sequencing type, completed genome sequencing, and retrieval of NBCI genome data. This search should be more suited for beginners.

### Stay informed - inform others

To stay up-to-date with newly sequenced genomes without repeatedly accessing diArk we offer an RSS-feed. To easily inform others, diArk offers options that allow the user to send content to facebook-, twitter-, and email-accounts.

## Results and Discussion

**Figure 4:**
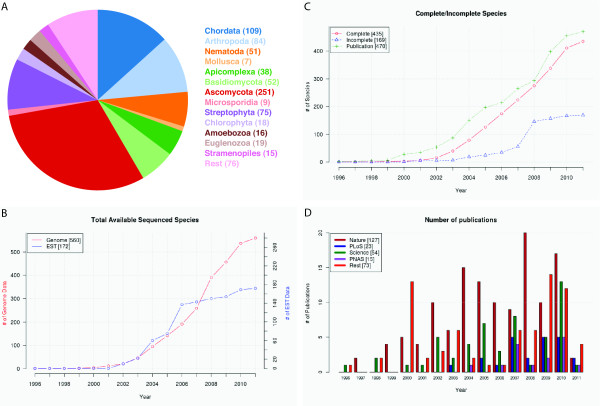
**Eukaryotes sequenced worldwide**. A) The pie chart shows the sequenced species sorted by taxa for which genome assemblies have been released. B) The graph shows the increase of total sequenced eukaryotes, genome data as well as EST data, in dependence of the year. Note that the lower numbers in the figures compared to the numbers given in the text are due to the fact that dates, at which genomes had been made available, are not known for every genome assembly. C) The graph shows the sequenced eukaryotes separated according to complete and incomplete (low-coverage genomes) genome assemblies. In addition, publications of genome assemblies are plotted. D) The diagram shows the number of publications of genome assemblies separated to four major publishing groups, the Nature Journals, the PLoS Journals, Science, and the Proceedings of the National Academy of Science (PNAS).

diArk is the most comprehensive and complete database for eukaryotic sequencing projects. The number of sequenced species and projects has more than doubled since the first version of diArk went online (Figure 4, [19]). diArk now (March 2011) contains 806 species (415 in 2007; numbers in parenthesis refer to database content in 2007), of which 613 (209) were subject to whole genome sequencing. Genome sequence data is referenced by 1911 (824) species project pages that are organized into 101 (73) sequencing centres.. The number of sequenced species is not as strongly increasing as might have been expected (Figure 4B). The discrepancy between the expected sequencing throughput and the only slightly exponential increase of sequenced species is best explained by the increased use of next-generation sequencing machines for other projects then de-novo sequencing of eukaryotes, like for human sequencing in the course of the 1000 Genomes Project [7] and for metagenome projects, which are not covered by diArk. Also, most likely due to next-generation sequencing the number of incomplete genomes (genomes sequenced with very low coverage) does not increase as strongly as before (Figure 4C). The strong increase between 2007 and 2008 is due to the low coverage sequencing of more than 60 Saccharomyces strains [36]. Although some sequenced genomes are awaiting analysis and publication since years, most genome sequences are published shortly after their generation (Figure 4C). The genomes of most sequenced species are still published in the high-impact journals Science, those of the Nature group, PNAS, and the PLoS journals (Figure 4D).

### Taxonomic distribution

**Figure 5:**
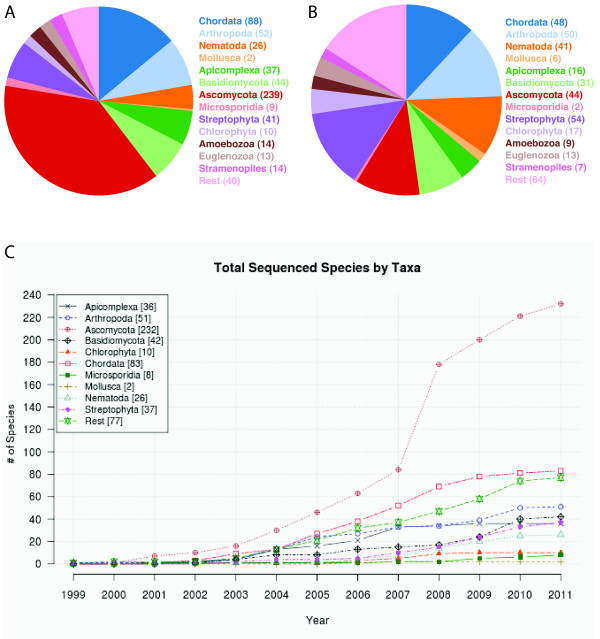
**Species sequenced in relation to taxa**. A), B) The pie charts show the number of sequenced species ordered by several major taxa. Graphs were drawn separately for species A) whose genome was sequenced and B) for which transcriptome data is available. C) Species are plotted according to the year in which the first genome assembly has been released. The species are combined to the same taxa as in A) and B).

As in 2007, whole genome sequencing is still strongly biased towards sequencing of fungi (especially ascomycotes) and chordates (Figure 5A). However, in 2007 we pointed out [19] that sequencing of nematodes and plants is far underrepresented, and this has changed dramatically. The number of sequenced nematodes and plants increased five fold in the last years while the number of the other sequenced species doubled to tripled (Figure 5C). The taxonomic distribution is still better balanced for transcriptome sequencing (Figure 5B).

### Sequencing methods

**Figure 6:**
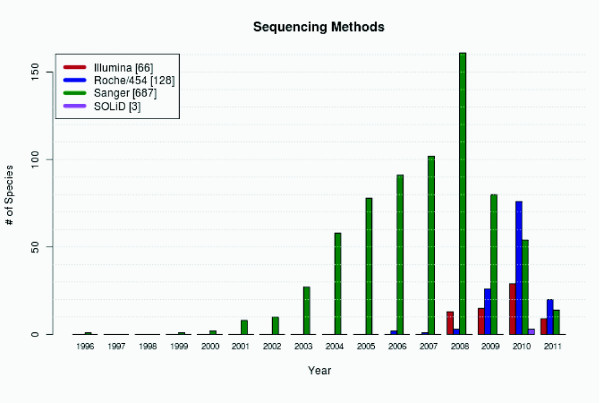
**Number of species sequenced by a certain sequencing method per year**. The diagram shows the number of species sequenced with different sequencing methods. For species that were sequenced using several methods (e.g. the whole genome library was sequenced with 454 and the BAC library sequenced with Sanger), every method is counted.

Since the first sequencing of a genome using massively parallel DNA sequencing [37] the Sanger method has increasingly been substituted by the high-throughput methods Roche/454, Illumina Solexa, and SOLiD (Figure 6). These methods pose several restrains to de-novo species sequencing like the need for a far higher sequencing coverage (some species like *Oreochromis niloticus *are sequenced with a coverage of more than 200 using Illumina) and specific assembly software. Both characteristics have been included in diArk.

### Genome characteristics

**Figure 7:**
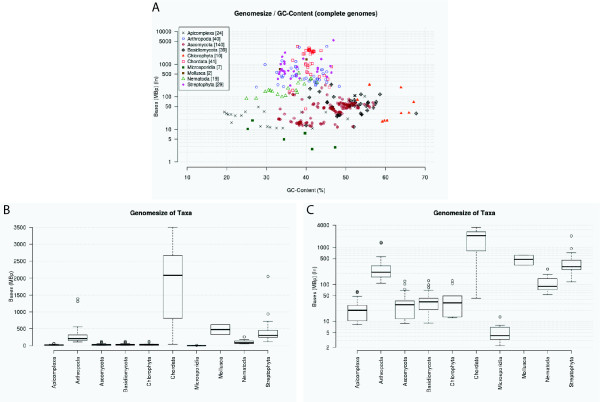
**Genome assembly characteristics**. A) The graph shows the GC-content and the genome size of completed genome assemblies (thus excluding low-coverage genomes). For better visualisation the genome size is plotted logarithmically. B) The diagram shows the box plot of the genome sizes of some major taxa for which many completed genome assemblies are available. C) Same as B) but the genome sizes are plotted logarithmically to better visualize the sizes of the smaller genomes.

Based on the genome assembly files diArk calculates several genome assembly characteristics like the number of contigs, N50 values, GC-content, and genome size. The plot of the genome sizes of completed genome assemblies against their GC-content shows taxa specific distributions (Figure 7A). Chordates have the largest genomes (and also a wide distribution of genome sizes, Figure 7B) but a narrow distribution of their GC-contents between 37-47%. Apicomplexa have the broadest distribution with GC-contents ranging from 20-55%, while Chlorophyta have the highest GC-contents (52-67%).

### diArk in comparison to other databases

Important parameters describing diArk's content in comparison to that of GOLD, NHGRI, NCBI Genome, and ISC are listed in Table 1. Because diArk, NHGRI, and ISC exclusively contain eukaryotes only those data were compared. Most obviously, the total number of species differs by up to a factor of ten. At diArk, information about 806 species is available (numbers have been obtained on March 10, 2011) while GOLD provides data for 2153 eukaryotes with 1876 species unique. NHGRI lists 187 (total 248), NCBI Genome 986 (total 1090), and ISC 287 (total 360) unique species, respectively. In total, GOLD and NCBI Genome list more species than diArk, but this is mainly due to the different philosophies. GOLD and NCBI Genome include species for which genome projects are planned or which are in very early stages ("DNA received" or "sequencing in progress") of the project while diArk only lists projects for which genome assemblies or considerable amounts of cDNA/EST data are available. In addition, GOLD, NHGRI, NCBI Genome, and ISC list the same species multiple times if for example different sequencing centres sequence different genome libraries (e.g. three entries are available for sequencing *Bos taurus *at GOLD), while diArk combines these data. Different strains of a species (e.g. *Saccharomyces cerevisiae YS2 *and *YS4*) are treated separately in all databases. Thus, the up-to-dateness of the databases can only be compared at the level of draft, finished, and published genomes. In diArk, 613 of 806 species are completely sequenced and 358 are published. In contrast, GOLD assigned 358 of the 2153 species as completed and 156 as published genomes. Publications for species are missing in GOLD for example (chosen alphabetically) for the pea aphid *Acyrthosiphon pisum *[38], the giant panda *Ailuropoda melanoleuca *([39], still marked as "in progress"), the fungus *Ajellomyces capsulatus NAmI WU24 *[40], the American malaria mosquito *Anopheles darlingi *([41], still marked as "in progress"), and the fungus *Ascosphaera apis *[42], while the list of 156 "published genomes" also contains species marked as "unpublished" (e.g. *Arthroderma benhamiae*) and those, for which no information at all is given (e.g. the four *Arabidopsis thaliana *ecotypes Bur-0, C24, Ler-1, and Kro-0). At NCBI Genome, 431 completed and 285 published eukaryotes were found. Because species projects and publications are entered manually into diArk and the other databases, the lower numbers by GOLD and NCBI Genome might mainly result from oversight and lack of manpower by the curators. diArk includes all publications listed in GOLD and NCBI Genome. Furthermore, diArk is unique in providing additional information for most of the sequenced genomes like the method(s) used for sequencing, the method(s) used to create the assembly, and assembly details like the sequencing coverage or the assembly version. For each assembly, the GC-content and the assembly size are computed while NCBI Genome and GOLD provide these data for only a small subset of their species. Based on these data, diArk presents the most comprehensive and complete dataset of sequenced eukaryotic species worldwide.

**Table 1:** diArk's content in comparison to other databases

	diArk	GOLD	NHGRI	NCBI Genome	ISC
# species (unique/total)	806	1876/2153	187/248	986/1090	287/360
# mRNA sequencing projects	562	350 (EST) 88 (Transcriptome)	11 (RNA) 1 (cDNA)	-	6 (cDNA) 1 (EST)
# genome sequencing projects	1499	1705	160	1078	-
# genomes marked as "sequenced" ^1)^	613	358 (completed)	88 (completed)	431	105
# genomes marked as "published" ^2)^	358	156	-	285	-
taxonomy	full taxonomy	two major taxa	one major taxon	two major taxa	one major taxon
sequencing method	✓	-	-	-	-
assembly method	✓	-	-	-	-
GC-content (# species)	589/613	142/1876	-	-	-
genome size (# species)	589/613	510/1876	-	✓	-
assembly details	✓	-	-	-	-
genome assembly files analysed	2109	-	-	-	-
species common names	✓	✓	✓	-	✓
links to species pages	✓	✓	-	-	-
detailed info about species pages	✓	-	-	-	-
sequencing centre reference	✓	✓	✓	✓	✓
funding agency	-	✓	✓	-	✓
target (survey sequencing, draft, etc.)	-	-	✓	✓	✓
project status	-	✓	✓	✓	✓
database search options	✓	✓	-	limited	limited
database content view options	7 result tabs	1 table	1 table	1 table	1 table
accessibility/speed	fast	slow	fast	fast	fast

^1) ^In this analysis, all genomes, for which assemblies were announced, are regarded as "sequenced" independently of the various status that the different databases give (draft, completed, published) and independently of the genome coverage.

^2) ^The numbers of published genomes have been retrieved as follows: **diArk**: 1) Using the Search page, select Projects_Search_Module, select "Sequencing type" Genome, and "Select all references" All Projects; 2) Add Search_Module, select Publications_Search_Module, and select "Select all publications" All Publications. **GOLD**: The number of published genomes is given, separated by kingdoms, in the "Complete Published" list. **NCBI Genome**: The number of published genomes has been derived by counting the links to PubMed.

NHGRI: http://www.genome.gov/10002154 (acquisition of data: 2011-03-10)

NCBI Genome Projects: http://www.ncbi.nlm.nih.gov/genomeprj, http://www.ncbi.nlm.nih.gov/genomes/leuks.cgi (acquisition of data: 2011-03-10)

ISC: http://www.intlgenome.org/viewDatabase.cfm (acquisition of data: data as of 2011-03-10)

## Conclusions

Due to the next-generation sequencing methods genome data of eukaryotes is increasing rapidly. Technically, all methods have their advantages and disadvantages, and it is therefore important to know how the genome of interest has been sequenced. Also, different assemblies have been generated for several species using either the same raw data but different assembly methods [33,34,43], or incorporating data from different sources (see for example the latest *Rattus norvegicus *assembly, version 4.1, generated at the Human Genome Sequencing Center at Baylor College of Medicine). diArk stores all genome assemblies that are available worldwide and provides several assembly related metadata: assembly version, assembly release date, completeness of the assembly, GC-content, assembly size, number of contigs, N50-value (including graphical representation of the contig distribution), accession numbers of the contigs, genome assembly files, sequencing method, and assembly method. diArk also provides many statistical analyses of its content based on the selection of the data. Currently, diArk contains data associated to 806 species. For 611 of them, genome assemblies are available, in most cases in different versions and types (contigs, supercontigs, chromosomes, etc.) amounting to 2109 genome assembly files. Of these 611 genome assemblies, 358 have already been published. Compared to other databases diArk 2.0 provides the most recent and comprehensive eukaryotic genome assembly data.

## Availability and Requirements

Project name: diArk - a resource for eukaryotic genome research

Project home page: http://www.diark.org/

Operating system: Platform independent

Programming language: Ruby

Other requirements: The current version of diArk was designed for Firefox, but has been tested on all recent versions of Safari, Internet Explorer, and Chrome. It requires cookies and JavaScript enabled.

License: The database schema, the web application and all scripts can be obtained upon request and used under a GNU General Public License.

## Competing interests

The authors declare that they have no competing interests.

## Authors' contributions

MK specified the requirements from a user's perspective, defined the rules for data handling, and collected all the data. BH and FO designed the database scheme and set up the technical requirements. BH, FO, and MH did the technical design and the programming. MK and BH wrote the manuscript. All authors read and approved the final manuscript.

## Supplementary Material

Additional file 1 **Database scheme. The file contains the detailed database schema**. Click here for file
