# Supplementary material for: diArk 2.0 provides detailed analyses of the ever increasing eukaryotic genome sequencing data
Source: BMC Res Notes. 2011 Sep 9;4:338. doi: 10.1186/1756-0500-4-338 (PMC3180467; doi:10.1186/1756-0500-4-338)
Supplement: Additional file 1 — Database scheme. The file contains the detailed database schema. [file 1756-0500-4-338-S1.PDF]

Figure 1. Database schema.

The schema shows the database tables and their relations. Content related tables are grouped and colored according to Figure 1 of the main manuscript. For each table the columns are listed with their name and datatype. Yellow keys in front of the names signify columns with unique identifiers. Blue window-symbols mark foreign key columns that contain values of id-columns of other tables. Symbols at the right side of the column names designate indices for better performance. Lines are relations between tables. Two unary (recursive) relationships are defined: One linking taxa to their parent taxon and one linking species groups to their parent group.

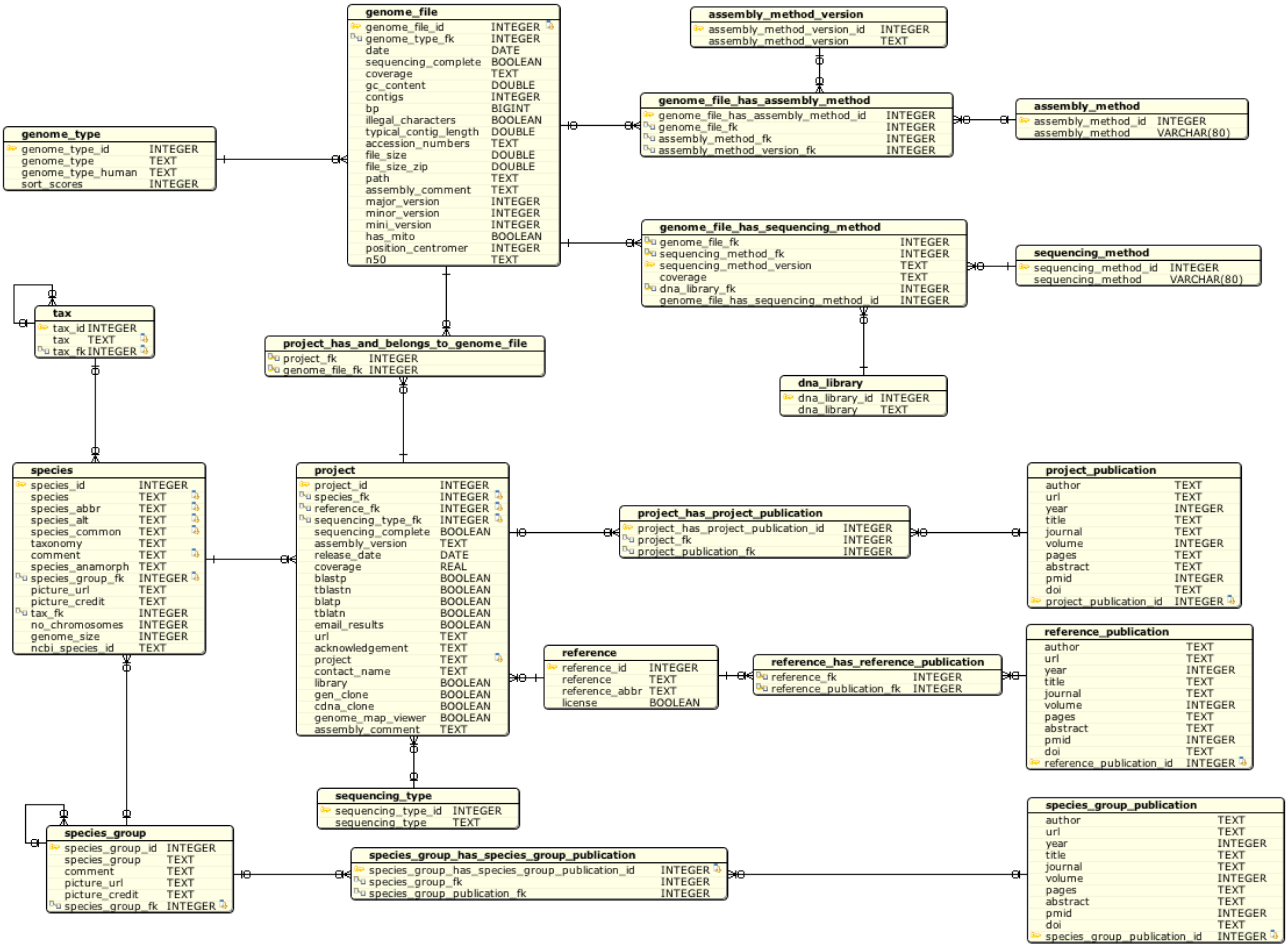
genome_file
genome_file_id INTEGER
genome_type_fk INTEGER
date DATE
sequencing_complete BOOLEAN
coverage TEXT
gc_content DOUBLE
contigs INTEGER
bp BIGINT
illegal_characters BOOLEAN
typical_contig_length DOUBLE
accession_numbers TEXT
file_size DOUBLE
file_size_zip DOUBLE
path TEXT
assembly_comment TEXT
major_version INTEGER
minor_version INTEGER
mini_version INTEGER
has_mito BOOLEAN
position_centromer INTEGER
n50 TEXT
assembly_method_version
assembly_method_version_id INTEGER
assembly_method_version TEXT
genome_file_has_assembly_method
genome_file_has_assembly_method_id INTEGER
genome_file_fk INTEGER
assembly_method_fk INTEGER
assembly_method_version_fk INTEGER
assembly_method
assembly_method_id INTEGER
assembly_method VARCHAR(80)
genome_type
genome_type_id INTEGER
genome_type TEXT
genome_type_human TEXT
sort_scores INTEGER
genome_file_has_sequencing_method
genome_file_fk INTEGER
sequencing_method_fk INTEGER
sequencing_method_version TEXT
coverage TEXT
dna_library_fk INTEGER
genome_file_has_sequencing_method_id INTEGER
sequencing_method
sequencing_method_id INTEGER
sequencing_method VARCHAR(80)
tax
tax_id INTEGER
tax TEXT
tax_fk INTEGER
project_has_and_belongs_to_genome_file
project_fk INTEGER
genome_file_fk INTEGER
dna_library
dna_library_id INTEGER
dna_library TEXT
species
species_id INTEGER
species TEXT
species_abbr TEXT
species_alt TEXT
species_common TEXT
taxonomy TEXT
comment TEXT
species_anamorph TEXT
species_group_fk INTEGER
picture_url TEXT
picture_credit TEXT
tax_fk INTEGER
no_chromosomes INTEGER
genome_size INTEGER
ncbi_species_id TEXT
project
project_id INTEGER
species_fk INTEGER
reference_fk INTEGER
sequencing_type_fk INTEGER
sequencing_complete BOOLEAN
assembly_version TEXT
release_date DATE
coverage REAL
blastp BOOLEAN
tblastn BOOLEAN
blatp BOOLEAN
tblatn BOOLEAN
email_results BOOLEAN
url TEXT
acknowledgement TEXT
project TEXT
contact_name TEXT
library BOOLEAN
gen_clone BOOLEAN
cdna_clone BOOLEAN
genome_map_viewer BOOLEAN
assembly_comment TEXT
project_has_project_publication
project_has_project_publication_id INTEGER
project_fk INTEGER
project_publication_fk INTEGER
project_publication
author TEXT
url TEXT
year INTEGER
title TEXT
journal TEXT
volume INTEGER
pages TEXT
abstract TEXT
pmid INTEGER
doi TEXT
project_publication_id INTEGER
reference
reference_id INTEGER
reference TEXT
reference_abbr TEXT
license BOOLEAN
reference_has_reference_publication
reference_fk INTEGER
reference_publication_fk INTEGER
reference_publication
author TEXT
url TEXT
year INTEGER
title TEXT
journal TEXT
volume INTEGER
pages TEXT
abstract TEXT
pmid INTEGER
doi TEXT
reference_publication_id INTEGER
sequencing_type
sequencing_type_id INTEGER
sequencing_type TEXT
species_group
species_group_id INTEGER
species_group TEXT
comment TEXT
picture_url TEXT
picture_credit TEXT
species_group_fk INTEGER
species_group_has_species_group_publication
species_group_has_species_group_publication_id INTEGER
species_group_fk INTEGER
species_group_publication_fk INTEGER
species_group_publication
author TEXT
url TEXT
year INTEGER
title TEXT
journal TEXT
volume INTEGER
pages TEXT
abstract TEXT
pmid INTEGER
doi TEXT
species_group_publication_id INTEGER
